# Books and Pamphlets Received

**Published:** 1877-07-20

**Authors:** 


### BOOKS AND PAMPHLETS RECEIVED.

PROCEEDINGS AND REPORTS OF THE SANITARY COMMISSION OF THE CITY OF ATLANTA, GA., 1876.

The reports contained in this publication are interesting and instructive, and evince thorough research and careful investigation.

The reports of Dr. Rauschenberg and Goldsmith constitute full and elaborate expositions of the two prominent methods for the removal of excreta, etc., from cities. These papers should be read by medical men, containing, as they do, information relating to health and medical science, in a department seldom studied and but little understood.

While we concede much ability and interest to the paper of Dr. Rauschenberg, we specially commend the report of Dr. W. T. Goldsmith, our associate editor, as presenting an able and interesting review of the whole field of sanitary reform, as drawn from the experience and observation of leading and advanced minds in Europe and America.

TRANSACTIONS OF THE MEDICAL SOCIETY OF THE STATE OF CALIFORNIA.

Through the kindness of our medical friend, H. S. Orme, M.D., of Los Angelos, we have a copy of the above interesting compilation.

After an able and entertaining address, by A. B. Nixon, M.D., President, we note able and interesting papers by the following medical gentlemen: Drs. Gibbons, Cushing, Montgomery, Shurtleff, Dubois, Wilder, Wells, Cox, Woolsey, Regensberger, Miller, and Wenzell. We regret that the Transactions reach us too late for a more particular notice at this time. It is a neat and creditable document of 168 pages, and speaks well for the profession in the young State of California.

*Popular Science Monthly*, for July, comes to us filled with instructive scientific articles, exceedingly interesting both to the intelligent popular reader and to the progressive medical man. It is, indeed, an ably conducted and valuable periodical.

USE OF LARGE PROBES IN THE TREATMENT OF STRICTURES OF THE NASAL DUCT. By Samuel Theobald, M. D., surgeon to Baltimore Eye and Ear Dispensary.

THE WOODRUFF SCIENTIFIC EXPEDITION AROUND THE WORLD. Containing organization, plan, management, etc., of a contemplated expedition to be assisted and encouraged by the government, to start in October, 1877, and return in October, 1879.

Dr. Hammond, of New York, has in preparation "On the Influence of the Maternal Mind on the Offspring During Pregnancy and Lactation." By William A. Hammond, M. D., Professor of Diseases of the Mind and Nervous System, in the Medical Department of the University of the City of New York.

In this work the author discusses the influence exerted by the mother during pregnancy and lactation on the physical moral, and mental characteristics of her offspring. A notable feature is the many points relating to ante-natal education; for Dr. Hammond believes, and facts appear to warrant the conviction, that it is before birth that the training of the child should begin.

*Solution and Absorption of Medicines, or the Best Means of Securing the Good Effects of Medicine on the Cure of Diseases—Read before the Tri-States Medical Society, at Vincines, Indiana, by J. W. Compton, M. D., Professor of Materia Medica and Therapeutics in the Medical College of Evansville, Indiana.* The subject of this paper is one of great practical importance.

